# Medical Schools in the Provinces

**Published:** 1919-09-06

**Authors:** 


### MEDICAL SCHOOLS IN THE PROVINCES.

**Birmingham University (Faculty of Medicine).**—In order to obtain the degrees of M.B. and Ch.B. five examinations have to be passed. The first is in chemistry, physics, and elementary biology; the second in anatomy and physiology; the third in general pathology, bacteriology, and materia medica and pharmacy; the fourth in forensic medicine, toxicology, public health, therapeutics, and special pathology; and the fifth, or final, in medicine, surgery, midwifery, diseases of women, mental diseases, and ophthalmology. The University also grants degrees and a diploma in dental surgery, and a diploma (D.P.H.) and a degree in Public Health (B.Sc.). The General Hospital and the Queen's Hospital, containing between them over 500 beds, are amalgamated for the purpose of clinical instruction, which is carried on under the direction of the University Clinical Board. Medical students also have free access to clinical instruction at the Eye, Ear and Throat, Orthopædic and Spinal, Women's, and Children's Hospitals, which are associated with the University. The composition fee for the whole course of lectures and instruction at the University is £85, besides which there is a composition fee for attendance on the medical and surgical practice and the clinical lectures at both the General and Queen's Hospitals of £42, making a total of £127. Dean: Professor Peter Thompson, M.D. The winter session opens on Tuesday, October 7, 1919.

**Bristol University.**—The University grants the conjoined degrees of M.B., Ch.B., the degrees of Doctor of Medicine (M.D.), Master of Surgery (Ch.M.), Bachelor of Dental Surgery (B.D.S.), and Master of Dental Surgery (M.D.S.)*; also diplomas in Public Health (D.P.H.), in Dental Surgery (L.D.S.),* and in Veterinary State Medicine (D.V.S.M.). For the conjoined degrees of M.B., Ch. B., the curriculum occupies five years and a half. The University lectures and laboratory courses are attended in the large and well-equipped new wing of the University. Hospital practice and clinical instruction are provided in the allied hospitals, which together afford upwards of six hundred beds and a very large external

* The fee for laboratory instruction in mechanical dentistry has been reduced to 40 guineas, which covers two years' instruction.

midwifery department. Three years at least must be spent in the University, and two of them subsequently to passing the second examination. Women are admitted to all degrees and diplomas and to the courses of study necessary for them. The winter session opens on October 1, 1919.

**Cardiff University College.**—Since it was founded in 1883, University College, Cardiff, has prepared students for the First Medical examination of the University of London, and for the corresponding examinations of other licensing bodies. Subsequently Chairs of Anatomy, Physiology, and Pathology and Bacteriology, and Lectureships in Materia Medica and Pharmacy and Histology and Embryology were established, making it possible to spend at least three out of the five or six years of the medical curriculum at this school. The Medical and Surgical degrees of the University of Wales are preceded by either the B.A. or the B.Sc. of that University. Arrangements have been made with the managing committee of King Edward VII.'s Hospital, Cardiff, to give students of the college the privilege of attending this hospital, which contains about 320 beds. The composition fee for the three years' course of study for the First and Second Medical examinations of the University of London is £63.

**Durham University.**—For students who intend to graduate at Durham University it is necessary that one of the five years of professional education should be spent in attendance at the College of Medicine, Newcastle-upon-Tyne. During the year so spent the student must attend lectures at the College and the medical and surgical practice and clinical lectures at the Royal Victoria Infirmary, which contains 600 beds. No residence at Durham is necessary, the Medical Faculty being in Newcastle-upon-Tyne. The remaining four years of the curriculum may be spent at Newcastle-upon-Tyne or at one or more of the recognised medical schools. The composition fee for the complete course of lectures is 90 guineas, and for the medical and surgical practice of the hospital 35 guineas. There are four professional examinations for the M.B., B.S. degrees. Fees, £30. Address, Professor Robert Howden, The College of Medicine, Newcastle-upon-Tyne.

**Leeds University (School of Medicine).**—This University grants the degrees of M.B. and Ch.B., M.D. and Ch.M., M.Ch.D. and B.Ch.D., and, in association with the Universities of Liverpool, Manchester, Sheffield, and Birmingham, holds a Matriculation examination. Clinical instruction is given in the General Infirmary, which contains 620 beds, and is amply provided with special departments. Students of the University also have access for instruction to the Leeds Public Dispensary, the City Fever Hospitals, the Hospital for Women and Children, the Leeds Maternity Hospital, and the West Riding Asylum at Wakefield. Diplomas in Public Health, Psychological Medicine, and Dental Surgery are also granted.

**Liverpool University (Faculty of Medicine).**—The University grants degrees in Medicine (M.B.; M.D.), in Surgery (Ch.B.; Ch.M.), and in Hygiene (M.H.). Candidates for degrees are required to pass the Matriculation examination or an equivalent. Students may study in the University for the degrees or for the examinations of other licensing bodies. The laboratories are modern and very well equipped, and the facilities for instruction are most complete. Courses of instruction in tropical diseases can be obtained at the Liverpool School of Tropical Medicine. Fellowships, scholarships, and prizes of over £1,000 are awarded annually, in addition to entrance scholarships. The Clinical School of the University now consists of four general hospitals—the Royal Infirmary, the David Lewis Northern Hospital, the Royal Southern Hospital, and the Stanley Hospital; and of five special hospitals—the Eye and Ear Infirmary, the Hospital for Women, the Infirmary for Children, St. Paul's Eye Hospital, and St. George's Hospital for Skin Diseases. These hospitals contain in all a total of about 1,134 beds. The organisation of these hospitals to form one teaching institution provides the medical student and the medical practitioner with a field for clinical education and study which is unrivalled in extent in the United Kingdom. The University also grants a licence and degrees in dental surgery, a diploma and a degree in veterinary medicine, a diploma in public health, and a diploma in tropical medicine. The Dental Hospital was opened in January 1910, and its equipment for teaching purposes is complete.

**Manchester, Victoria University (Faculty of Medicine).**—The medical department of the University, which is open to both men and women students, is particularly well provided with facilities for instruction in all the courses for degrees and the professional qualification and for teaching the preliminary subjects—namely, anatomy, physiology, pathology, materia medica, biology, physics, and chemistry. Clinical instruction is provided at the Manchester Royal Infirmary, which contains 882 beds, and also has large out-patient and casualty departments. Associated with the Infirmary are a Convalescent Hospital at Cheadle, containing 136 beds, and the Royal Lunatic Hospital, accommodating 430 patients. Clinical instruction in gynæcology is also given at St. Mary's Hospital, and other hospitals.

**Sheffield University (Faculty of Medicine).**—The degrees of the University, M.B., Ch.B., M.D., Ch.M., and the diploma in public health are open to men and women alike. A candidate for the degrees of M.B., Ch.B. must produce certificates that he will have attained the age of 21 years on the day of graduation; that he has pursued the courses of study required by the University Regulations during a period of not less than five years subsequently to the date of his matriculation or exemption from matriculation, three of such years at least having been passed in the University, one at least being subsequent to the passing of the first examination. The University is within easy reach of the various hospitals with which it is connected for clinical purposes. The composition fee for University lectures, practical classes, and hospital practice is £30 a year for each of the five years. The University of Sheffield offers many valuable scholarships and prizes to students in the faculty of medicine.

